# The influence of the hidden curriculum on the risk of burnout in junior doctors in a palliative medicine rotation – a qualitative exploratory study

**DOI:** 10.1186/s12904-025-01665-x

**Published:** 2025-02-12

**Authors:** Eng Koon Ong, Ranitha Govindasamy, Wen Shan Sim, Lalit Kumar Radha Krishna

**Affiliations:** 1 Division of Supportive and Palliative Care, National Cancer Centre Singapore, 30 Hospital Boulevard, Singapore, 168583 Singapore; 2 Division of Cancer Education, National Cancer Centre Singapore, Level 21, 30 Hospital Boulevard, Singapore, 168583 Singapore; 3 Duke-NUS Medical School, National University of Singapore, 8 College Rd, Singapore, 169857 Singapore; 4 Assisi Hospice, 832 Thomson Rd, Singapore, 574627 Singapore; 5 Yong Loo Lin School of Medicine, National University of Singapore, 1E Kent Ridge Road, NUHS Tower Block, Level 11, Singapore, 119228 Singapore; 6 Department of Maternal-Fetal Medicine, KK Women’s and Children’s Hospital, 100 Bukit Timah Road, Singapore, 229899 Singapore; 7 Antenatal Risk Assessment Unit, KK Women’s and Children’s Hospital, 100 Bukit Timah Road, Singapore, 229899 Singapore; 8 Centre for Biomedical Ethics, National University of Singapore, Blk MD11, 10 Medical Drive, Singapore, #02-03, 117597 Singapore; 9 Palliative Care Institute Liverpool, Academic Palliative & End of Life Care Centre, Cancer Research Centre, University of Liverpool, University of Liverpool, 200 London Road, Liverpool, L3 9TA United Kingdom; 10 The Palliative Care Centre for Excellence in Research and Education, PalC c/o Dover Park Hospice, 10 Jalan Tan Tock Seng, PalC, Singapore, 308436 Singapore

**Keywords:** Junior doctors, Palliative care, Death and dying, Moral distress, Hidden curriculum, Burnout

## Abstract

**Background:**

Palliative Care (PC) provides person-centred care for patients with life-limiting diseases and their families. Studies have shown that healthcare professionals delivering PC are predisposed to moral distress and burnout due to constant exposure to death and dying and aspects of the hidden curriculum (HC) through which culture and values are transmitted implicitly. However, there are limited studies focusing on the latter through the lens of junior doctors. Using the Ring Theory of Personhood (RToP) and the Krishna-Pisupati Model (KPM), which categorize and map conflicts between personal and professional values, beliefs, and principles within the four domains of personhood, this study investigates the impact of palliative care experiences on the risk of burnout in junior doctors.

**Methods:**

This qualitative exploratory study was conducted at the Division of Supportive and Palliative Care in the National Cancer Centre Singapore, involving medical residents who had completed at least one month with the division between 2020 and 2022. 13 participants were recruited for individual semi-structured interviews carried out by an independent research assistant. Acknowledging HC and burnout as sociocultural constructs, we adopted a constructivist ontological position and a relativist epistemological lens to guide thematic analysis of the data.

**Results:**

The themes identified were: (1) The Nature of PC (2), Moral Distress, and (3) Impact of Environment on Wellbeing. Junior doctors saw the value of the philosophy of care in PC and felt compelled to adopt values espoused by the discipline. However, compounded by consistent exposure to death and dying and limitations to manpower and time, elements of the HC, such as staff support measures, proved to be significant stressors—contributing to burnout and moral distress.

**Conclusion:**

This is the first study on the effects of the HC on burnout for junior doctors in a PC rotation. It provides unique insights into the impact of complex clinical, personal, social, ethical and organizational factors on burnout and suggests that all factors need to be addressed in tandem for any attempts at staff support to be successful. This study can guide current and future research and programs that support wellbeing for junior doctors.

**Supplementary Information:**

The online version contains supplementary material available at 10.1186/s12904-025-01665-x.

## Introduction

Palliative Care (PC) seeks to improve the quality of life for patients with life-limiting diseases while also providing support to their families [1]. In doing so, PC teams often face emotional, existential, moral and ethical distress [2–5]. Even as data tells us that between 24% and 33% of local Palliative Care Physicians (PCPs) experience burnout, these concerns are often given short shrift [6, 7]. Moreover, the exact proportion of junior doctors in PC suffering such symptoms are worryingly absent [8–10]. Given that the perceived incidence of burnout and distress is higher among PCPs [11, 12] due to prolonged exposure to death and dying [13, 14], the impact of a PC posting on junior doctors is worth exploring. In addition, our literature review on PCPs and burnout elucidated the need to consider wider cultural and systemic factors within PC, including the effects of battling bureaucratic red tape [15], a lack of resources [16] and limited support [6, 7, 12, 17–20].

The consideration of the hidden curriculum (HC), *“a set of influences that function at the level of organisational structure and culture including*,* for example*,* implicit rules to survive the institution such as customs*,* rituals*,* and taken for granted aspects”* [21] is therefore essential. The HC has been increasingly cited in the literature as an important consideration for burnout [22–24] and shines the spotlight on environmental working factors, rather than focusing on the ability of individuals to develop resilience. The effect of the HC on burnout has largely been negative, with consequences of a negative HC including a loss of idealism, emotional erosion, increased cynicism, ethical dilemmas, moral distress and burnout [10, 23, 25–29]. Consequently, poorly supported HCs limit the recruitment of residents into residency programs [24].

However, various authors have suggested that the unique nature of person-centred care amongst patients at the end-of-life in PC may counter burnout [13, 30–32]. These authors also argued that the HC in PC has helped PCPs to find meaning in their work and achieve better job satisfaction. Consequently, the HC in PC has also been credited with lowering burnout rates for physicians. Such a perspective has not been reported in other specialities, suggesting that the effect of the HC is likely to be discipline-specific. Current literature on the HC within PC is narrative in nature and does not investigate the impact of the HC on burnout as its primary research question—presenting an urgent gap in data as we cope with the struggles of the pandemic and the moral, emotional and ethical distress in daily practice [33–37].

## Context and environment of practice

PC practice in Singapore revolves around hospital and community services, including inpatient care in acute hospitals and community hospitals, inpatient hospices, home hospice and day-care hospices [33]. This study focuses on the experiences of medical residents attached to the Division of Supportive and Palliative Care (DSPC) at the National Cancer Centre Singapore (NCCS), Singapore’s quaternary cancer centre. DSPC provides three specialist consult teams for patients admitted to Singapore General Hospital (SGH). Each team consists of two PC specialists, a senior resident or resident physician, two or more junior doctors and a PC nurse who, together, provide PC advice, recommendations and guidance on compassionate discharge, hospice placements and follow-up plans with other care teams, such as oncology, respiratory and renal medicine, cardiology, gastroenterology, surgery, geriatrics and intensive care.

Medical residents who are attached to the DSPC teams would have completed at least one year of their residency training in SGH’s Accreditation Council for Graduate Medical Education International (ACGME-I)-guided Internal Medicine residency training program and have elected to be attached to PC as part of their 3-month elective.

### Theoretical framework

A constructivist ontological and relativist epistemological lens is adopted to study changes in the somato-psycho-social-semiotic perspectives [38, 39] within current concepts of self-concepts of personhood and identity.

**Fig. 1:**
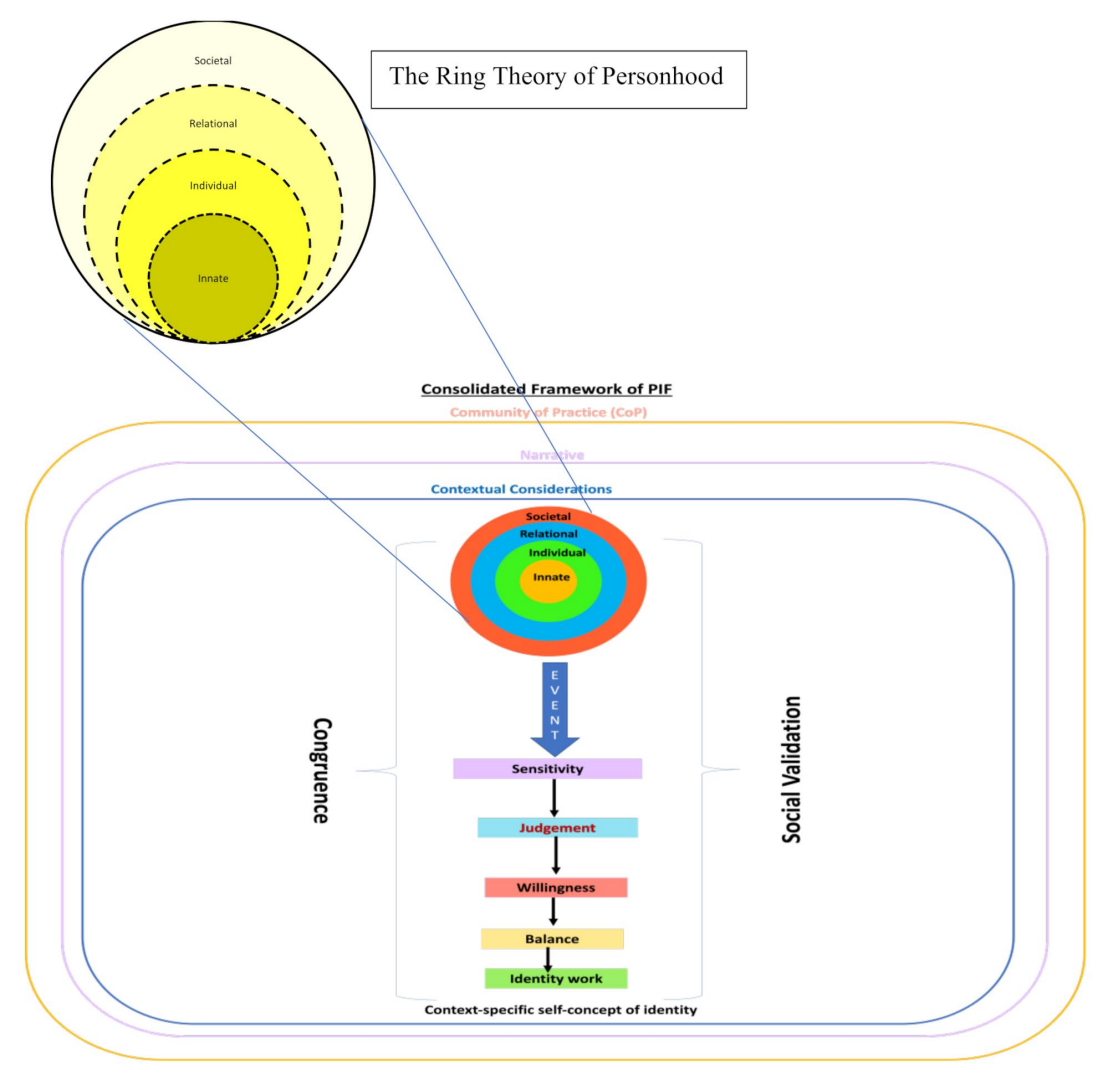
The Krishna-Pisupati Model of Professional Identity Formation [40]

This lens also allows for the use of the Krishna-Pisupati Model (KPM) [40] to map the impact of these experiences on a junior doctor’s identity (Fig. 1). The KPM is built around the Ring Theory of Personhood (RToP) [41], which suggests that values, beliefs and principles (belief systems) define an individual’s self-concepts of personhood and identity. The RToP suggests further that the four domains of Innate, Individual, Relational and Societal personhood and identity contain domain-specific belief systems within the four rings of the RToP. Changes in one or more of these belief systems will result in changes in self-concepts of identity and personhood. The intertwined nature of different aspects of identity, relationships, the working environment and nature of PC conditions and patients underpins our combined study of the HC and burnout. These changes in belief systems begin when new beliefs, values, principles, expectations and considerations (collectively ‘life experiences’) are introduced. The integration of new and current belief systems changes self-concepts of personhood and identity and shifts thinking and conduct [41–43]. We explain the key terms in the KPM in Table 1 for ease of review.

**Table 1:** Elements of the Krishna-Pisupati Model (KPM)

Term	Meaning
Belief system within the Innate Ring	Religious and cultural beliefs, moral values and ethical principles
Belief system within the Individual Ring	Autonomous function and individual characteristics
Belief system within the Relational Ring	Values governing personal relationships
Belief system within the Societal Ring	Belief system guiding peripheral relationships and societal, professional and legal expectations
Resonance	New life experiences are consistent with the physician’s values, beliefs and principles
Synchrony	Resonant values, beliefs and principles are reprioritized to better fit within practical considerations
Dissonance	New life experiences clash with existing belief systems
Disharmony	Dissonance within one ring of the RToP
Dyssynchrony	Dissonance between rings of the RToP
Sensitivity	Detection of resonance, synchrony, disharmony and/or dyssynchrony
Judgement	Physicians determine whether a response to an event is required
Willingness	Physicians consider their motivation, ability and capacity to adapt their identity in response to an event
Balance	The prioritization of these adaptations to preserve identity
Identity work	The process through which physicians adapt their identity

### Research question

Guided by the literature review, this qualitative study employs individual semi-structured interviews with junior doctors within DSPC. The study is guided by the research question: How do junior doctors in a palliative care rotation experience the hidden curriculum?

## Methodology

### Participant characteristics

This study was designed to adopt a qualitative exploratory approach. Medical residents within DSPC who have completed at least one year of residency training and at least one month of their rotation in PC were included in the target population. They will henceforth be referred as junior doctors in relation to their level of training and the need for supervision by senior PCPs. They are expected to acquire basic competencies that include performing comprehensive PC assessments encapsulating symptom assessments, psycho-emotional and social dimensions of care and evaluation of the patient’s family and caregiver needs and coping. In addition, junior doctors are also expected to provide good symptom control through initiation and titration of opioids, conduct of conversations about goals of care with patients and their next-of-kins, contribution during complex case discussions and provide psychosocial and bereavement support. During their postings, junior doctors must also expand their interprofessional and communication skills, working hand-in-hand with the rest of the PC team which comprise nurses, counsellors, social workers and therapists.

To help them achieve these learning objectives and acquire the requisite competencies, junior doctors are furnished with case discussions, bedside teaching and interactive lectures on topics like symptoms assessment and medication use, communication skills and ethical reasoning and uses. Learning is also driven by role-modelling, guided immersion, facilitated reflections in team debriefs, interdisciplinary communication and the culture and HC within DSPC. Achievement of these learning goals is determined largely through formal, informal and multisource feedback from the team on their general performance.

### Recruitment of participants

Junior doctors who met the above inclusion criteria between January to December 2022 were invited to participate in this study while other junior doctors, including doctors on other attachments, overseas visitors and fellows, registrars and resident physicians, were excluded. The recruitment period was extended to include January 2020 to December 2021 to improve the response rate. A total of 63 junior doctors were invited and 13 participants responded to the invitation email and were successfully recruited from this group.

Participants who provided written consent to the study were verbally consented again prior to their individual hour-long semi-structured interviews with an independent research assistant (RA). In view of prevailing pandemic measures, the recruitment and consent-taking process were completed semi-electronically according to institution protocols. The initial contact with the participants was done through email by the principal investigator where a summary of the study was provided. A reminder email was sent again in two weeks for non-respondents. Contact made through emails ensured that pressures to respond due to time and physical location of the participants were avoided. Participants who responded were then sent the Participant Information Sheet and consent form in electronic softcopies, based on the template provided by the local institutional review board. These summarised the study design and potential benefits and risks. Electronically signed consent forms were returned via email and stored by the RA under password encryption. Participants were then contacted by the RA for details regarding the interviews, which were conducted online for convenience. Other recruitment strategies included regular presentations at departmental teachings and administrative management meetings to increase awareness of this study and garner support from senior management to release junior doctors from clinical duties for participation in the individual interviews.

### Data collection

Data collection and data analysis took place concurrently. Recruitment was stopped when thematic saturation was achieved or when dominant themes were repeated, with no new insights from two subsequent interviews. Individual interviews elicited deeply personal experiences of the participants through recall and rich descriptions [45] and were selected as the method of data collection in this study. Focus group discussions (FGDs) might have allowed deeper interactive sharing of common experiences of the HC [46] but the personal nature of these experiences meant that discussions might have been curbed by FGDs. Similarly, surveys were not used as they were more suitable for collecting quantitative data and did not allow for ‘deep dives’ into the responses provided [12, 47, 48]. A semi-structured interview with pre-determined, open-ended questions allowed focused yet flexible discussions about the HC and burnout [49]. This was important to ensure a rich collection of data, even when participants had limited understanding of the HC.

The data was audio-recorded on the online meeting platform and the RA anonymized the transcriptions for data analysis. Participants were assigned numbers (P1, P2, P2…) by the RA during data analysis. The semi-structured interviews employed a mix of prompts and direct questioning and were constructed around data drawn from my literature review (see supplementary file 1). The transcripts were stored electronically with password encryption.

### Rigor of data collection

The RA was a non-clinician who was trained by the study team prior to the start of participant recruitment on interview techniques and contextual information, such as the approach to PC, roles of junior doctors, medical hierarchy and reporting structure, current healthcare and medical education systems, research methodology and ethics and concepts of the HC and burnout. Practice interviews were carried out and the interview guide was piloted with two junior doctors from the same target population to increase familiarity and participant comfort before formal interviewing took place. The Hawthorne effect [50] was considered but review of the audio-recordings did not suggest that participants modified their responses due to a sense of being observed by the RA. The use of unobtrusive online platforms for the interviews also ameliorated the impact of the Hawthorne effect. Triangulation of the data collected was not possible due to a single data source. However, data collected was returned to the participants to ensure accuracy of transcription (member checking) by the RA.

### Data analysis

Thematic analysis, a qualitative research method that *“recognises, investigates and categorises patterns within data”* was used [51]. This method often goes further to interpret and provide new insights into different perspectives of the study. A reflexive approach ensured that data analysis was iterative, open and flexible [52, 53]. As the coding process progressed, interpretation of the data evolved and strengthened the conceptualization of the themes.

The process of data analysis started with the creation of familiarization notes using Microsoft Excel (2018). The transcripts of all the participants were read in an analytic manner and with an open mind. Points of interest were noted and the initial codes guided by the research question were generated [54]. This involved collating points in the familiarization notes to reflect and interpret what was shared by the participants. The list of codes was then reviewed as part of the creation of a code book. Together with field notes, the reproducibility and auditing of the analysis were enhanced.

The initial themes were created to consolidate common understanding and patterns within the codes with main anchor concepts. Through an iterative process that led to repeated reflections on the codes and editing of the themes, an early thematic map was created. The thematic map was finalized after further iterative reflections on the data and initial themes and decisions on suitable names that were descriptive and concise. Together with individual subthemes supported by the relevant and impactful codes, the results of the data analysis will be presented in the next section.

## Results

A total of 63 junior doctors were invited to participate. Semi-structured interviews were conducted with five male and eight female junior doctors, aged between 26 and 38 years old who were between three to eight years postgraduate. Three main themes were identified: (1) the nature of palliative care (2) moral distress, and (3) the impact of the environment on wellbeing.

### Nature of palliative care

The participants described the clinical care they provided in PC and how they tried to honor its philosophy of care. Primarily seeing patients who required symptom management and were dying, spending more time getting to know the patient as a person and building a relationship with them defined the nature of PC for the junior doctors and distinguished PC from other medical rotations. They identified a link between the nature of PC and their wellbeing.

#### Types of patients and conditions

Participants struggled with issues inherent to death and dying, including symptom management and difficult conversations:

*“*…*finding it difficult to manage his pain because he was wilfully—he wanted to be in a state of pain because he perceived that that’s his deserved state so it was very difficult because he was refusing all his painkillers and they were asking us for help.”* (P8).

*“…there were a lot of difficult conversations taking place and at the end of the week, I was just wondering why was I so tired… even though I didn’t actually have to work overtime.”* (P7).

*“Many people die every week… we do see that in other departments but in pall(iative care) a lot more…”* (P9).

One of the junior doctors felt that burnout was secondary to the nature of PC:

*“I feel that the intrinsic burnout just comes from the nature of the work itself and not so much from the hidden curriculum.”* (P12).

Participants reflected on how PC was practised in an acute tertiary medical centre and a quaternary centre:

*“More fast-paced… a lot more symptoms management in very aggressive ways. There are a lot of interventions available to treat your symptoms and there are always a lot of re-evaluations, re-scans and everything, blood tests and all that. So, a lot of the patients we see are not necessarily very conservative … also very varied kind of cases you see all kinds of patients.”* (P6).

#### Philosophy of care

PC’s philosophy of care that focused on comprehensive person-centred care was clearly evident in its practice and clinical approach. To realize PC’s comprehensive person-centred care approach, participants reported actively participating in deeper conversations and forming closer ties with patients, resulting in the development of more personalized interactions than those they had in previous postings:

*“Spending more time with the patient… getting to know the patient as a person and… understanding… who they live with… hobby*,* spiritual beliefs…” (P9)*.

While developing these relationships allowed junior doctors to better understand and attend to the personalized needs of their patients, the deep ties formed also made witnessing the eventual deterioration of their patients difficult:

*“It’s quite hard… I had built a relationship… I struggled with this most during the posting.”* (P11).

*“So*,* you—because usually in palliative medicine you will actually ask about the patients and because we need to know more about the patients, sometimes you will feel quite sad for the patient because of their unfortunate experience, which makes me quite sad and actually affects my own feelings.”* (P1).

P3 and P8 reported that there was a lack of time to acculturate to the PC approach, setting and practice, which were different from other disciplines, and to align expectations:

“*…we didn’t expose ourselves to it previously and now you realize that you need to use a lot of this (psychosocial aspects) in order to … speak to patients and to better understand them. I guess that’s where the learning curve is…”* (P3).

*“…every home team is different. They have a different culture*,* different expectation*,* different people*,* different mood*,* different combination of personalities and they will expect the visitor to get to know them and to kind of integrate into that culture. But for me, being the one who is being rotated over and over and over again, it’s very difficult…”* (P8).

Compounding feelings of grief from the demise of their patients they had built a relationship with were the long hours, meeting complex needs, attending family conferences and delving into the psychosocial aspects of care and discharge planning. These factors caused moral distress when junior doctors were not able to achieve the outcomes that PC advocated for, elaborated upon in the next section.

### Moral Distress

The participants struggled with moral distress when limited manpower and time resources competed with the aspirational standards of care. One example was the role-modelling by senior faculty who invested considerable time and effort in clinical communications:

*“Irrespective of how difficult the communication scenario was or how much time it would take*,* they would invest that effort into doing this for the patient or family and if I take a step back and ask why… I think there is … dedication to that craft and being there for the patient and family… It is inspiring to see someone who has been in this field for very long and still able to do something with so much passion…”* (P11).

Consequently, when the junior doctors found it difficult to balance between task completion and the aspired goals of PC practice, a sense of guilt, helplessness and moral distress surfaced, particularly when service standards superseded the values-based standards espoused by PC:

*“…it becomes a lot work-focused than… from a genuine place of care… we all care to a certain extent it’s just that… Get the job done… your boss expects it*.” (P6).

*“I mean we all care to a certain extent, it’s just that sometimes when you’re so tired and stretched*,* you don’t have the capacity to care a 100% for every person.” (P5)*.

“*There was one particular patient actually which trigged that emotion because… he passed away very suddenly in hospital and it just felt like whatever we were doing for him didn’t really help… I was quite affected by that*.*”* (P7).

The struggle to balance service needs with time and manpower constraints further influenced how the working environment impacted well-being. This is elaborated in the final theme.

### Impact of the environment on wellbeing

The junior doctors shared about how their experience of both formal staff support programs and informal interactions with their seniors corresponded to aspects of the HC which, in turn, led to a negative impact on their wellbeing. For many, the staff support sessions, which included art-making and small group sharing, ate into the working hours—leaving them scurrying to complete their clinical work at the end of the day. This added unnecessary stress and ironically compromised efforts to promote better work-life balance:

*“I think the intentionality and the heart behind it is there… But referrals and everything they still need(ed) to be seen… we have to get the work done*,* we want to go home.”* (P6).

*“*…*but I think like because you’re actually doing it during your workday*,* a work afternoon*,* I feel like it’s kind of counterintuitive because they want you to take part to show that they care about you but then there’s actually work and the work never ends… Someone in the back end is actually trying to mop up all the work for you so it feels (like)… time could have been better allocated because if we do it during work hours and there’s still task that needs to be done, then actually someone is suffering. So, there’s no welfare for the people who are doing the work while you are kind of having a nice session of weaving.”* (P2).

Compounding their misgivings, participants felt *“obliged to participate”* to fit into the team culture:

*“I think the implicit thing is that you should participate*,* and you should go for it… a few times that I was reminded about it… it gives me the feeling that, oh you know, I am expected to be there and I’m expected to sign up for it… I definitely felt pressured [slight laugh] to go for it maybe because you’re … one of the lowest in the whole organization [slight laugh] so if you are told to go then you should.”* (P12).

*“Actually quite stressful because sometimes we attend these things and there’s this implicit hint hint that you need to participate actively and [laughing] and sometimes you are just tired and you’re just like*,* ‘why am I even here?’ and ‘I don’t really feel it you know’ and I’m not very good at art.”* (P6).

Amidst these concerns about the formal staff support program, these sessions were met with disbelief for some participants and cynicism for others:

*“…how do you drop everything for an afternoon to sit there? I think the intention behind it is good and I appreciate it but [laughing] realistically not too sure about that?”* (P5).

*“I am quite cynical so the message I get is um they are doing these things because they gotta show that they are doing something.”* (P10).

Participants were also disappointed by the mixed messages by senior staff who appeared to prioritize personal wellbeing and advocated for better work-life balance when general clinical work still took priority:

*“…kind of like*,* ‘guys try not to—can you try not to…’ I do think that adds to burnout… you don’t really have a choice. You have to bring your work home.”* (P5).

Even when concerted efforts were made to reduce the hierarchy in the PC team where*“the team is a lot flatter in the sense that the seniors are a lot more approachable and accessible…”* (P11), alongside regular bonding sessions over meals and open discussions (P3) where *“you can talk to about these challenging conversations or emotions”* (P11), the disconnect between the practices being advocated and what was practiced and expected of the participants was evident:

*“You need to come early and you need to go back late. As I mentioned because… your own thing—you need to use your own time to do the—like to understand what the patient this kind of thing… So*,* although… official (working) hours but we will never—I will never actually get back on time…”* (P1).

A further example of this disconnect between the practices being advocated and practice expectations on the participants was seen in mortality meetings. P9 reported that even when mortality meetings were seen as opportunities to reflect upon a case, *“it’s still… very medical case presentation, as compared to really talking about the emotions that you are feeling.”* This sentiment was echoed by P10: 

*“Yah… a bit like a brush off but then I guess I don’t know whether… they are just used to it… so it really doesn’t— like they don’t really mean it in a mean way.”* (P10).

Similarly, the notion that PC espouses access to timely and personalized support was challenged as the reality of the situation was quite different:

*“To a large extent my bosses were not there… it was very very stressful but it’s just that my bosses are very busy.”* (P5).

Despite the above, participants were hesitant to provide honest feedback to their seniors as they feared misinterpretations of their intent and the potential repercussions:

*“…*,* it may not look very collegial for you to bring that up and you’re probably scared that there is no anonymity in bringing this kind of thing up and of course it just seems mean-spirited.”* (P4).

*“…*,* you would be afraid of how you will be graded or like in dealing with such things yah… Because every posting will hurt your grading…”* (P9).

## Discussion

In answering its research question, “How do junior doctors in a palliative care rotation experience the hidden curriculum?”, this semi-structured interview-based study provides new insights to current literature about how junior doctors cope in a PC posting. PC’s philosophy of care; the tension between values-based goals and service-based requirements; and how staff support was experienced are all important influences which are further explored below.

First, our data suggests that the nature of PC was more likely a risk factor, rather than a protective factor, towards burnout despite other authors suggesting the contrary [30–32]. As with practice in family medicine [56], geriatrics [57] and paediatrics [58], PC’s use of time, effort and personal investment in building personal connections with patients and their families [56, 59] has been purported to promote meaning-making and enhance physician job satisfaction [12, 30–32, 60, 61]. However, our participants also suffered from encountering deaths of patients they had developed a deeper understanding of [6]; the “culture shock” from moving from “traditional” medical postings to PC [30]; and vicarious trauma attending to patient’s hurt, despair, loss and death. This is despite their willingness to provide the person-centred care espoused in PC as they saw the value of their work towards patient care. In investigating how such stressors may be countered, various authors have studied the adequacy of palliative care training at undergraduate and postgraduate levels in preparing students and junior doctors in managing PC patients [61–63]. However, without a common agreement about learning objectives, consistent assessment methods and longitudinal mentorship, these authors report similar struggles—supporting the need for further research to address these gaps.

Exacerbating the above, the participants reported struggling with moral distress stemming from the inability to fulfill service standards in the face of limited manpower and time resources. Such standards superseded the values-based standards of PC and may be considered as an important component of the HC as this tension was rarely addressed explicitly. Instead, participants were left with a sense of helplessness, guilt and an apparent lack of support from their seniors who were seen as facing similar struggles.

Similar reports of moral distress, or being forced to act against personal or professional values, or in an unethical manner to achieve clinical requirements [27, 64–67] due to an organizational climate poorly equipped to support the individual needs of junior doctors [66] and accommodate all stakeholder needs and conditions have been reported elsewhere. Harrison et al. [27] and Epstein and Hamric [65] highlighted the importance of organizations in taking responsibility to address moral distress while the association between moral distress and burnout is further demonstrated in internal medicine residents by Lamiani et al. [66] and Sajjadi et al. [67]. Quek et al.’s [68] scoping review on moral distress in physicians reiterates the need for an individualized means of supporting physicians at risk. This appears to be lacking currently for our participants. Within PC, other authors have noted challenges with balancing service standards [69–71] and aspirational PC standards [1, 8, 73]. We believe that our study is the first to focus exclusively on junior doctors and on how this phenomenon affects junior doctors through the HC, where such a disconnect was strongly felt and rarely addressed.

Finally, the experience of the environment in terms of staff support appears to be the most affected by the HC. The existing staff support programs within the palliative care department are largely based on the arts and humanities and small-group sharing [8–10]. Positive outcomes in the acceptability and feasibility of employing literary works and visual art pieces to facilitate discussions on challenging clinical encounters and difficult conversations have been previously published [8–10]. However, despite best intentions, these programs were seen as no more than distractions and often regarded with a mixture of cynicism and scepticism [23, 74, 75]. Chuang et al. suggest that support in navigating professional dilemmas may prevent negative perceptions about staff support. The authors propose a five-step strategy to address this through processes like self-reflection, staff interviews and surveys and data analysis [23]. Shapiro et al. [74], through the lens of undergraduate medical education, echoed the importance of addressing moral distress and wellbeing to support the relevance of staff support programs. Benbassat [75] listed four undesirable features of the learning environment that contradict staff support efforts, including a climate of fear in making errors, a denial of uncertainty in outcomes, abuse of junior staff, prejudice against mental illness and a reluctance to seek help. These insights mirror the data of our study and may explain why participants felt implicitly coerced to attend staff support programs and that the use of the arts to platform debriefs and share challenging clinical scenarios were ineffective [9, 75–80].

In addition, the impracticality of well-intentioned messages of support from seniors suggests that attempts to muster the team together amidst staff shortages and increased work stressors should be re-evaluated on practical grounds [82]. It is apparent that junior doctors, although appreciating such verbal expressions of support, may ironically become demoralized and frustrated when actual support does not eventually materialize. There is also a risk of disenfranchisement and distrust when seniors are perceived as only providing “lip service”. However, none of the participants reflected back to their seniors about the negative impact of such remarks. Although the reason for not doing so was not available in our data, this may illustrate how the HC is particularly powerful as a risk factor for burnout due to its inherent implicitness. Indeed, our data suggest that when poorly planned [83, 84] and accompanied by informally enforced attendance, staff welfare programs had the opposite effect on team-building and wellbeing, especially when the HC was not addressed.

### Junior doctors in palliative care – A particularly vulnerable group?

Our data raises several areas of concern about the risk of burnout in junior doctors in PC. Junior doctors have been reported to be subjected to various unique stressors, including inherently difficult clinical scenarios and experience, long working hours, time demands and interpersonal interactions at work [8–10, 85]. We also posit that the brevity of rotations decided by national needs means that junior doctors may not have the opportunity to develop resilience and coping strategies that senior palliative care doctors achieve after years of experience in PC. This observation has been shared by those in Internal Medicine and other PC institutions [86, 87]. Short rotations may also result in limited interactions with senior PC physicians, little opportunity for debriefs [77], insufficient time and opportunity to build relationships and seek mentoring support [88, 89]. Secondly, authors have suggested that the PC approach focusing on person-centred care may naturally result in a relatively rare instance of a *positive* HC towards its junior doctors [30, 90]. It is possible that with such expectations, junior doctors who experience the contrary when they rotate into PC may be particularly frustrated, confused and cynical [91]. Finally, perhaps the biggest concern stems from the apparent lack of awareness by senior PC physicians regarding their contribution towards a negative HC despite best intentions to provide a supportive and non-hierarchical working environment for the junior doctors. It is unclear if this was a result of senior PC physicians feeling a need to portray a professional identity of compassion and empathy towards junior doctors to reflect concordance in how PC is practiced [91–93]. Further research is needed in this aspect urgently to avoid further entrenchment of such a HC within PC.

### Limitations

This study is limited by the small sample size and a single setting, which may restrict the generalizability of these findings beyond a local Singapore hospital perspective, replete with its unique practice and structure. The recruitment period was initially slow and had to be extended. Similarly, the enrollment rates were low, although this may be in view of concerns over psychological safety and an apparent negative perspective of the posting. This could be due to the intense but brief nature of the posting, leaving most junior doctors emotionally and physically tired and unmotivated to participate, nor willing to reflect on their experiences [95]. The retrospective nature of data collection may thus have led to some considerations and meaning-making being lost during recall. Finally, the input and perspectives of other stakeholders, such as other senior doctors and allied health professionals within PC, are also absent. Future studies should consider a longitudinal approach towards data collection that spans multiple sites and settings involving other stakeholders that influence the HC and its impact on burnout.

## Conclusion

This is the first study that sheds light on the effects of the HC on burnout for junior doctors in a PC rotation. It provides unique insights into the impact of complex clinical, personal, social, ethical and organizational factors on burnout and suggests that all factors need to be addressed in tandem for any attempts at staff support to be successful. This study can guide current and future research and programs that support wellbeing for junior doctors.

## Electronic supplementary material

Below is the link to the electronic supplementary material.

Supplementary Material 1

## Data Availability

No datasets were generated or analysed during the current study.

## Declarations

### Animals’ involvement

This study does not involve animals subjects.

## Abbreviations

ACGME-I: Accreditation Council for Graduate Medical Education International
DSPC: Division of Supportive and Palliative Care
HC: Hidden Curriculum
KPM: Krishna-Pisupati Model
NCCS: National Cancer Centre Singapore
PC: Palliative Care
PCPs: Palliative Care Physicians
RToP: Ring Theory of Personhood
SGH: Singapore General Hospital
SEBA: Systematic Evidence-Based Approach
