# Supplementary material for: The influence of the hidden curriculum on the risk of burnout in junior doctors in a palliative medicine rotation – a qualitative exploratory study
Source: BMC Palliat Care. 2025 Feb 12;24:40. doi: 10.1186/s12904-025-01665-x (PMC11817082; doi:10.1186/s12904-025-01665-x)
Supplement: Supplementary file 1 — Supplementary Material 1 [file 12904_2025_1665_MOESM1_ESM.docx]

Interview guide

| Prompts | Probes |
| --- | --- |
| Q1. Can you describe your learning experience during your palliative medicine rotation? | |
| - What are the common learning activities during your rotation? | - Are there any learning points you picked up that were not formally taught? |
| Q2. What do you understand by the term Hidden Curriculum? | |
| - The hidden curriculum is defined as a set of influences that function at the level of organisational structure and culture, including implicit rules to survive the institution such as customs, rituals, and taken for granted aspects. | - Can you describe any such customs or rituals within the learning and working environment that you have observed? |
| Q3. What do you think is the impact of the Hidden Curriculum on your learning and working experience as a junior doctor in the department? | |
| - Can you describe how the hidden curriculum impacted you positively or negatively as part of the clinical team? | - Do you think the hidden curriculum resulted in mainly positive or negative influences for you? |
| Q4. As a junior doctor, how do you think the hidden curriculum impacted your mental wellbeing? | |
| - Do you think resilience or burnout are affected by the hidden curriculum? | = How does the hidden curriculum affect burnout risk for you? |
| Q5. How do you think the hidden curriculum affects other members of your team? | |
| - Do you think the hidden curriculum affects different team members similarly or differently? | - Does profession, seniority and appointments affect how the hidden curriculum impacts other team members? |

**Notes:**

- The general findings from this interview were used in the paper *The Impact of Experiences in Palliative Care on the Personal and Professional Development of Junior Doctors* submitted to BMC Palliative Care
- There are plans to publish another paper specifically detailing findings related to the Hidden Curriculum
